# Women’s experiences of their sexuality during their menopausal transition and the support offered to them by healthcare providers: A systematic review and meta-synthesis

**DOI:** 10.18332/ejm/209571

**Published:** 2025-09-18

**Authors:** Amanda Calvin, Sarah Cina, Ulrika Byrskog, Kerstin Erlandsson, Catrin Borneskog

**Affiliations:** 1 School of Health and Welfare, Dalarna University, Falun, Sweden

**Keywords:** support, menopause, healthcare, sexuality, perimenopause, informed choice

## Abstract

**INTRODUCTION:**

Menopause can significantly impact women's sexuality and sexual health, yet knowledge gaps among healthcare practitioners is a barrier to adequate support. Research on women's issues is less prioritized and limited funds are invested in studying female sexuality. In addition, women may feel hesitant to seek medical assistance for sexual health concerns during menopause due to lack of knowledge or societal taboos.

**METHODS:**

This is a meta-synthesis of qualitative research on women's experiences of their sexuality during the menopausal transition and the support offered by healthcare providers. Data collection was conducted using PubMed, CINAHL, and PsycINFO. A total of 21 qualitative studies from diverse cultural contexts, including Lebanon, Iran, Sweden/Chile, Spain, UK, Ireland, Thailand, Singapore, Australia, USA, China, Malaysia, and Taiwan, were synthesized, encompassing 610 participants.

**RESULTS:**

Women's experiences of sexuality during menopause are highly individualized and influenced by relationship dynamics, sexual autonomy, and personal perceptions of menopause. The synthesis also highlighted a common concern: women reported a lack of adequate healthcare support, knowledge, and targeted treatments to address their sexual well-being during this period.

**CONCLUSIONS:**

Menopause can profoundly affect women's health, sexuality, and quality of life. For women to make informed choices regarding menopausal healthcare, extended knowledge, education, destigmatisation, and access to healthcare are essential. This synthesis underscores the critical need for enhanced education and interdisciplinary collaboration within healthcare systems.

## INTRODUCTION

All individuals born with ovaries will go through menopause either naturally, surgically or medically. This article will refer to these individuals as ‘women’, although we acknowledge that not everyone will identify as such. Menopause is not explicitly mentioned in the Sustainable Development Goals (SDG)^1^, but considering its impact on women’s health, it could easily be argued as part of target 3 (good health and well-being), target 4 (quality education) and target 5 (gender equality). Despite the impact menopause has on women’s health and sexuality, it remains underrepresented, under researched, and taboo in many societies^2,3^. Besides the biological effects, gender and social norms can have an additional negative impact on women’s sexuality during the menopausal transition^4^. Women may experience the intersectional influence of both ageism and sexism as they grow older^5^. Wood et al.^6^ provide a broad synthesis of women’s lived experiences during the menopausal transition, offering valuable insights into sociocultural perceptions, access to care, and broader health-related concerns. Their narrative review identifies key challenges related to stigma, information access, and intersectional influences on menopausal health. Building on that, the present study expands to the dimension of sexuality and the role of healthcare support in this context. As such, this meta-synthesis offers a complementary but more targeted contribution to the existing literature.

Sexuality is a multifaceted phenomenon, shaped not only by biological factors but also by psychological, relational, and contextual elements and it is commonly affected by menopause^7^. As Nagoski^8^ emphasizes, sexual desire varies widely among women and is often context dependent. This perspective is essential in understanding women’s sexual experiences during menopause, as it challenges the notion that decreased libido is inherently pathological, instead situating it within a broader framework of sexual health and well-being.

Graugaard^9^ explains that there is a ‘two-way taboo’ with menopause, where neither patients nor healthcare professionals initiate conversations around the subject, which hinders an open communication and proper care from being provided. Lack of knowledge is an additional barrier to women’s healthcare; in a scoping review, Macpherson and Quinton^10^ argue that the way menopause is taught to professional healthcare workers and presented in textbooks is too simplistic to give a full understanding of the complex topic. In addition, it is estimated that approximately 49% of women do not feel informed at all about menopause before going through it^11^. A nationwide Swedish survey by Götze Eriksson et al.^12^ revealed significant disparities in menopausal hormone treatment (MHT) knowledge and prescribing practices between general practitioners (GPs) and gynecologist. GPs reported lower awareness of national guidelines and difficulty in prescribing MHT. Notably, nearly one-third of gynecologist also acknowledged insufficient education on MHT, suggesting that these knowledge gaps are pervasive across specialties.

According to Mirin^13^, funding for medical research is nearly twice as high for male medical issues compared to female ones. For example, extensive treatments are available for erectile dysfunctions^14^, while there is very little available for female sexual dysfunction (FSD). FDS is reported to affect 68–86.5% of menopausal women and 25–63% of premenopausal women^15^. FSD includes issues with libido, orgasm, arousal, and sexual pain as well as significant stress connected to these symptoms. It is often underdiagnosed and undertreated^16^. FSD may increase during menopause due to genitourinary syndrome of menopause (GSM). GSM is described by Portman and Glass^17^ as a collection of symptoms associated with decreased estrogen levels, affecting the labia, clitoris, vagina, urethra, and bladder. Symptoms may include dryness, burning, irritation, discomfort during sex, and urinary issues.

MHT has been shown to be an effective and safe treatment for many women, particularly when initiated within ten years of menopause onset. Current clinical guidelines from leading bodies such as the North American Menopause Society^18^, the British Menopause Society^19^ and the Swedish Society of Obstetrics and Gynecology^20^, support the use of MHT to alleviate menopausal symptoms. They state that benefits often exceed risks, and that improved long-term health outcomes are significant, including reduced risks of cardiovascular disease, osteoporosis, and GSM. Nevertheless, misconceptions and outdated fears about MHT’s risks persist among both healthcare providers and patients^12,21,22^. MHT can also have a direct positive effect on sexuality as it reduces and prevents GSM. As MHT increases women’s general wellbeing, it may also increase sexual interest indirectly. MHT does not, however, have a direct effect on libido and ability to orgasm^4^. There is an ongoing debate about testosterone for low libido in connection to menopause, but few countries have options adjusted to women’s dosage needs^7^.

In understanding women’s sexual experiences during menopause, it is essential to challenge the notion that decreased libido is inherently pathological and instead situating female sexuality within a broader framework of sexual health, relational health and well-being.

The aim of this study is to synthesize qualitative research on women’s experiences of their sexuality during the menopausal transition and the support offered to women by healthcare providers, using a meta-synthesis approach.

## METHODS

### Search strategy

**Figure 1:**
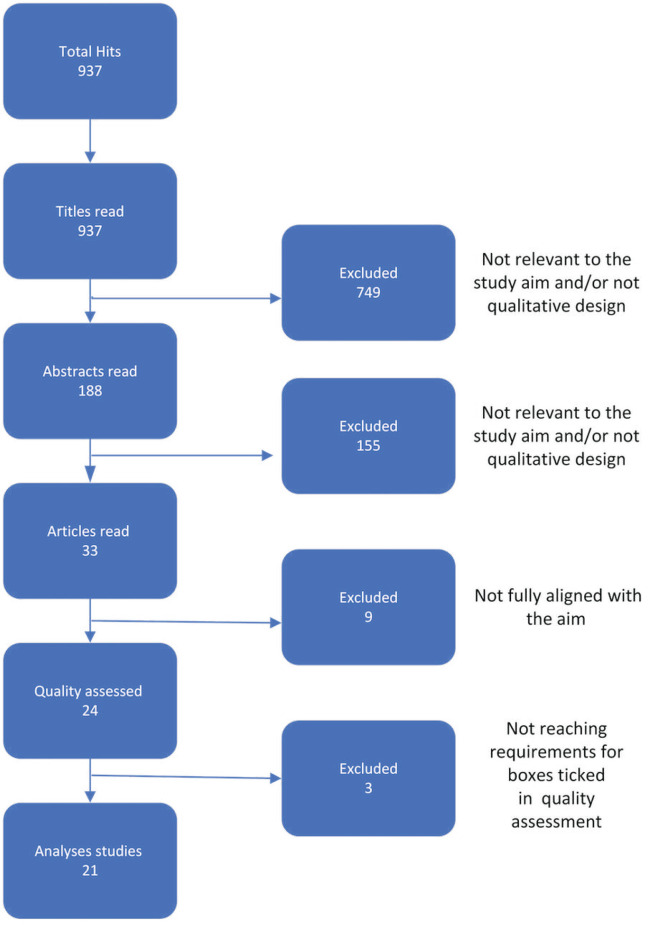
PRISMA flowchart, a meta-synthesis of sexuality during menopause, 2024

The search engines used for data gathering were PubMed, CINAHL and PsycINFO; searches were performed in January 2024. The key words used in searches were: ‘menopause’, ‘perimenopause’, ‘middle-aged woman’, ‘sex*’, ‘experience’, ‘quality of life’, and ‘qualitative’. Searches were limited to articles published after 2003, in English, and which were peer-reviewed. Boolean operators AND, OR, and NOT were used in different combinations with the search words to maximize the number of relevant hits. Expanded searches were made, but when using search words such as ‘support’, ‘healthcare’, and ‘nursing’, the hits received were on medical or surgical menopause, which were not relevant to the aim of this study. If the title seemed relevant to the aim, then the abstract was read and all articles with relevant abstracts were downloaded and read in full. The articles were checked against the inclusion criteria (Table 1) as well as whether they fit the aim of this study. Relevant articles were then quality assessed using the checklist for qualitative research from Joanna Briggs Institute (JBI). This review used PRISMA guidelines for reporting the search strategy. The search process yielded 937 studies which were screened for eligibility, 33 articles were assessed in full-text, 24 were quality assessed, and 21 articles were included (Figure 1).

**Table 1:** Search criteria, a meta-synthesis of sexuality during menopause, 2024

**Population**	Individuals, born with a uterus, who identify themselves as women, expressing their experiences of sexuality connected to their natural menopause
**Study design**	Qualitative or mixed-methods studies
**Type of article**	Academic, peer-reviewed research articles
**Publication language**	English
**Publication period**	2003–2024
**Quality assessment**	JBI critical appraisal checklist for qualitative research

JBI: Joanna Briggs Institute.

Studies with women who expressed their experience of sexuality connected to their menopausal transition were included. The 21 articles^23-43^ used in the synthesis had a total of 610 participants, aged 38–75 years, originating from Lebanon, Iran, Sweden/Chile, Spain, UK, Ireland, Thailand, Singapore, Australia, US, China, Malaysia and Taiwan (Table 2).

**Table 2:** Overview of included articles, a meta-synthesis of sexuality during menopause, 2024

*Authors* *Year* *Country*	*Aim of research*	*Method and analysis*	*Sample size* *Age (years)* *Menopause status*	*Key findings*
Amini and McCormack^23^ 2019 Iran	Examine menopause as an extended period during which women evaluate their lives in the context of the patriarchal culture of Iran.	Interviews Thematic and structural analysis	N=30 45+ Defined	Medicalized accounts of menopausal time and chaos narratives. Menopausal time and loss narratives. Finding agency in menopausal time.
Azar et al.^24^ 2021 Lebanon	Explore women’s perception and experience of sexual difficulties.	Interviews and focus groups Framework analysis	N=55 40–55 Not defined for all participants	Women’s inability to communicate sexual desires and concerns; male sexual difficulties; marital conflicts; and sexual difficulties as context bound. Women’s sexual difficulties are driven by double standards and inhibit sexual socialization.
Bahri et al.^25^ 2017 Iran	Explore the ways of managing sexual dysfunctions during the menopausal transition among Iranian women.	Interviews Content analysis	N=21 42–55 Defined	‘Adopting self-sacrifice’ Confronting decline of libido. Seeking strategies for coping. Achieving problem solving.
Binfa et al.^26^ 2009 Sweden Chile	Learn about Chilean women’s reflections about womanhood and sexuality during midlife.	Focus groups Thematic analysis	N=21 40–64 Not defined for all participants	Societal expectations on women, perceptions about sexual relationships, and women’s social stigmatization.
Dillaway^27^ 2005 USA	Will evidence of a change discourse and gendered beauty ideals appear within the interview data, and do these ideologies place women within a contradictory biosocial space? And, if so, how do women think and act toward their menopausal bodies?	Interviews and focus groups Thematic analysis	N=61 38–60 Defined	Menopausal bodies are changing bodies. Changing bodies are ‘undesirable and (therefore) invisible’. Controlling unruly menopausal bodies.
Dillaway^28^ 2005 USA	Describe how a sample of middle-class, heterosexual women discusses menopause as a reproductive and aging process and examines how menopause is distinct from other aging processes.	Interviews and focus groups Thematic analysis	N=45 38–60 Defined	Reproductive ageing no longer represents a symbolic end to reproductive capacity; more often it represents an end to contraceptive use and menstruation. Interviewees reported enjoying sex more than ever before because they could engage in this activity without the hassles of contraception and menstruation.
Dillaway et al.^29^ 2008 USA	Explore how privilege and oppression might determine the meanings and experiences of the menopausal transition.	Interviews and one focus group Thematic analysis	N=61 38–60 Defined	African American women and Chicanas viewed menopause as positive. Middle-class European American women were more negative. Women of color were more likely than European Americans to report talking about menopause with same-race, same-sex friends only. While women of color discussed European American women’s menopause, the former lacked knowledge of other women’s experiences.
Goberna et al.^30^ 2009 Spain	Describe women’s sexual experiences during the climacteric years.	Focus groups Content analysis and phenomenology	N=18 44–64 Defined	The climacteric stage, a time for self-reassessing; the burden of biology; the journey through sexual life; and the importance of social/family factors as regards sexual experience.
Hinchliff et al.^31^ 2010 UK	Examine women’s own experience of menopause.	Interviews Thematic analysis	N=12 48–60 Defined	Changes in sexual life: sexual desire, biological aspects of menopause, personal and social factors, interpersonal, orgasm, partners role, importance of sex, Impact on social wellbeing: distress, communication dissatisfaction, unmet sexual needs, adapt.
Hyde et al.^32^ 2011 Ireland	Present accounts of heterosexual women experience of sexuality in the context of menopause.	Interviews Thematic analysis	N=25 42–63 Defined	Discourse of biography The biomedical discourse Discourses of and discrepancies in sexual desire.
Javadivala et al.^33^ 2018 Iran	How women assign meaning to and process sexual motivation during the menopausal transition.	Interviews Content analysis	N=22 44–59 Defined	‘Diminished sexual capacity’ (effect of menopause, illnesses associated in mid-life, desire discrepancy); ‘Intimate coupling’ (lack of physical or emotional intimacy, couple communication and romance); ‘Sociocultural scripts’ (sexual script, parental responsibilities); and ‘Sense of youthfulness’ (having an active and happy life, maintaining physically attractiveness).
Moghasemi et al.^34^ 2017 Iran	Investigate Iranian women’s attitudes and experiences about sexual life changes in midlife.	Interviews Content analysis	N=17 40–65 Defined	Continuous paradox over being a sexual agent. Considering menopause; opportunities and threats for sexual life. Coping strategies for changes in sexuality in midlife
Noonil et al.^35^ 2012 Thailand	Understand and describe the lived experience of Thai women and their changing bodies in midlife.	Interviews Phenomenology	N=18 46–55 Defined	Changing in midlife. Sensing normal phenomena. Searching for explanation. Sense of loss. Self-managing.
Ong et al.^36^ 2020 Singapore	Increase the understanding of the experiences and needs of perimenopausal women with climacteric symptoms in Singapore.	Interviews Thematic analysis	N=20 47–54 Defined	Uncertainty and misconceptions Changes in the body. Mixed feelings. Social support. A ‘wish’ list of women.
Strezova et al.^37^ 2017 Australia	Explore the attitudes to, and experience of, menopause among Macedonian women living in Australia, including attitudes and responses to hormone therapy (HT) and complementary therapies, as well as related psycho-sexual, relationship and other midlife issues.	Focus groups Thematic analysis	N=81 45–75 Defined	Inadequate knowledge of menopause, symptoms of menopause, the use of HRT and alternative treatments, cultural issues (feeling culturally different), migration, employment, attitudes to menopause and associated psychological states, religion and fasting, impact of menopause on marriage, and the concept of the ‘male menopause’.
Thomas et al.^38^ 2017 USA	To better understand the sexual function outcomes that were most important to sexually active women aged 45–60 years and the types of treatments they would prefer.	Interviews and focus groups Thematic analysis	N=39 45–60 Not defined for all participants	Feeling attractive was an important reason for sexual activity. Changes in appearance, especially weight gain and breast changes, were common. Response to changes in appearance affected sexual satisfaction.
Thomas et al.^39^ 2018 USA	Understand midlife women’s lived experiences of changes in sexual function with aging.	Interviews and focus groups Thematic analysis	N=39 45–60 Not defined for all participants	The most common negative changes were decreased frequency of sex, low libido, vaginal dryness, and anorgasmia. Participants attributed negative changes to menopause, partner issues, and stress.
Thomas et al.^40^ 2019 USA	Explore how body image relates to sexual function and satisfaction in midlife women.	Interviews and focus groups Thematic analysis	N=39 45–60 Not defined for all participants	Women sought solutions to specific sexual problems: low desire, vaginal pain and dryness, and decreased arousal or ability to achieve orgasm. Women indicated emotional outcomes were most important to them. Women preferred behavioral over pharmaceutical treatments, citing concerns about side effects. Behavioral treatments might be better equipped to address physical and psychological aspects of sexual problems.
Wong et al.^41^ 2018 China	Explored the impact of menopause on sexual health and marital relationships, the associated factors and the support needed among middle-aged and older women.	Interviews Thematic analysis	N=30 43–64 Defined	Menopause had a considerable negative impact on women’s sexual lives. Vaginal dryness and low sex desire were most commonly reported. Knowledge financial support and family understanding were important in helping women manage menopause.
Wong et al.^42^ 2012 Malaysia	Explore attitudes toward midlife crises, experiences with midlife crises, help-seeking behaviors, and needs among multi-ethnic Malaysian women.	Focus groups Thematic analysis	N=89 45–75 Not defined for all participants	Concerns over physical aging and frequently reported experiencing midlife crises, triggered by issues such as empty nest syndrome and aging’s impact on various aspects of life. Comparatively less open attitudes toward sexuality and help-seeking for sexual problems were observed, while religious coping was frequently reported.
Yang et al.^43^ 2016 Taiwan	Examine Taiwanese women’s perspectives on the way menopause affected their sexual behavior to gain an in-depth understanding of their experiences during this transition.	Interviews Content analysis	N=18 45–60 Defined	Change in physical response during sex. Acceptance/non acceptance of current situation. Sexual pressure related to marital role. Efforts to improve sexual interest or activity.

### Data extraction and synthesis

An inductive meta-synthesis was conducted using a five-step procedure, as described by Malterud^44^. Step one was reading and coding the articles to determine their degree of relevance. This was done in parallel by two of the authors with supervision from the remaining authors. After assessing the data, a joint primary coding of the articles was done in NVivo^45^. Differences in interpretation were discussed until a consensus was reached. A second coding was then done in parallel where the articles were divided up and records were created of identified key themes, metaphors, and phrases in each separate article. Secondary key data and supporting primary quotations from participants in the included articles’ result sections were extracted (Table 3), all in line with the aim of this study^44,46^.

**Table 3:** Extraction of coding process, a meta-synthesis of sexuality during menopause, 2024

*Secondary key data*	*Supporting quote*	*Theme*	*Metaphor*
Several participants discussed how these beauty expectations were also policed by their husbands^23^.	Nahid, aged 51 years, described how a friend never smiled to reduce the build-up of wrinkles because her husband had instructed her to do this.	Appearance	Fertility, femininity and desirability
Some women believed that it was a wife’s responsibility to satisfy her husband’s sexual requests and thus they continued to have sex regardless of their own desires^41^.	*‘No interest … not aroused yet … I told him to be patient, (to go) slowly … more foreplay. If I did not let him (have sex), he then got (irritated) … He always thinks I use menopause as an excuse to not have sex.’*	Duty	Performative sex
Women can enjoy their sexuality more fully upon/ after reproductive aging, as they feel free from the gendered burdens of contraceptive use and monthly menstruation^28^.	*‘Menopause [is] wonderful. It’s like years ago, taking off your bra! No more Tampax!’*	Pregnancy/period	Menopause as sexual freedom
The most common negative changes were decreased frequency of sex, low libido, vaginal dryness, and orgasm difficulties^39^.	*‘Until you hit menopause, I don’t think you really realize what you’re facing when you cross over that bridge and you kind of always assume that your sex is going to be good … and then it changes and you’re like, “Really? This is what I’m stuck with for the rest of my life?”.’*	Symptoms of menopause	Rather no sex than bad sex
Belief that men had a biological urge to engage in sexual activity more so than did women^32^.	*‘And I was thinking to myself, “Is it only because we’re married that he feels he has these rights?”.’*	Discrepancy in sex drive	It takes two
Women’s sexual difficulties were mainly induced by an inhibiting sexual education and patriarchal marital relationships that favor men’s sexual satisfaction and rights^24^.	*‘The man sleeps with his wife and does nothing … You feel that you become used to that … He (her husband) enjoys it, and that is. It does not count if the woman enjoys or not. This is also my right, my right. If the situation was reversed … I do not think that he would have been patient.’*	Lack of education, lack of autonomy	Privilege matters
Perimenopausal women with climacteric symptoms had ‘wish’ lists that they hoped could be fulfilled, such as having more informational support, more understanding from peers and family, and empathy from healthcare professionals^36^.	*‘Be more attentive, be more delicate to us (sniffles) because we are the ones who need support, the ones that are actually going through [menopause].’*	Lack of healthcare	Support requires knowledge, will, access and acknowledgment

Step two was determining how the studies were related by identifying similarities and differences. Step three was translating the studies into a joint framework while keeping the essence of each separate study intact. Step four was synthesizing the translation, which was done by interpreting the data and looking for new insights. The themes were refined to create a synthesis with new conclusions and arguments for the overarching category and metaphors. Step five was expressing the synthesis, bringing the upcycled findings together and presenting the interpretations of the synthesized studies.

## RESULTS

We present the results of a meta-synthesis of 21 articles^23-43^. The participants of the studies expressed a large variety of experiences of their sexuality during their menopausal transition. The data show that sexuality is a complex phenomenon and that there are multiple factors at play in whether a woman is interested in remaining sexually active during and after her transition. The identified metaphors were: Fertility, femininity and desirability; Without autonomy sex is performative; Menopause as sexual freedom; Rather no sex than bad sex; It takes two; Privilege matters; and Support requires knowledge, will, access and acknowledgment. All of these metaphors can be summed up by the overarching theme: Sexuality during menopause is not a homogenous experience.

### Fertility, femininity and desirability

Women experience the loss of fertility during menopause differently. Some single women expressed that fertility itself was an attractive feature that men wanted in a partner, not connected to their desire to have children^27^. Other women expressed the loss of fertility associated with loss of femininity and the changes in appearance that menopause brought^23,27,37,40,43^. Some women associated hormonal changes in their cycle to sexual desire; with menopause these sensations in sexual desire were lost^27,31,32,36,43^:

*‘I don’t feel as attractive. I think people start to relate to me, not as a sexual being, but as a motherly person’*.^27^

Many women expressed difficulty accepting changes in their appearance; they talked about feeling a loss of womanhood as they went through the menopausal transition^23,26,27,35-37^. The primary physical changes expressed were weight gain, flattening breasts, facial hair growth and changes in skin tonus^23,27,28,35,40^. Many communicated that feeling good about themselves and how they looked made them more likely to view themselves as sexual beings or more motivated to engage in sexual behaviors^27,28,33,40^, while feeling bad about how they looked had the opposite effect^24,26,27,33,39,40^:

*‘Even 34 years later, sometimes I don't want him to see me naked. Now how sick is that? I’ve had two children with this man.’* ^40^

Some women expressed that their partners did not care about changes in their appearance^40^, while others felt pressured to go on diets, get beauty treatments and even have surgery^23,27,34,40^. But not all women struggled with their appearance; some expressed that with age they cared less about their looks, allowing them to enjoy sex more than ever^28,40^:

*‘I am much more open than I used to be sexually [because I] don't give a shit anymore. Sorry! (laugh) … I mean it’s like I’ve lived all my life for everybody else, right? So that part of it is really good. The part I have problems with is reaching orgasm. It used to be a whole lot easier.’* ^28^

### Without autonomy sex is performative

Performative sex is when the act is disconnected from desire and is instead based on gender expectations; women expressed that this stemmed from patriarchal societies, religious societies or from relationships with strong, explicit gender roles. They expressed this by describing sex as a duty or a chore that they were expected to participate in or felt responsible for^23-26,30-33,35,36,41,43^:

*‘I just look at sexual matters from a religious point of view… I accept everything for not being responsible in front of God. My husband likes having sex, but I don't. I try to meet his request I’m a bit afraid of God.’* ^25^

Some women expressed that they had never enjoyed sex^23,24,32,34^ and that going through menopause only made it different because it added more pain^23,34^. Some women worried that if they could not fulfil their husbands’ sexual needs, they would be useless in their role as a wife, or that their husbands would leave them or get a second wife or mistress^24-26,31-33,35-37,41,43^.

### Menopause as sexual freedom

Periods, contraceptives, and pregnancies all have a big influence on women’s sexuality. Because of this, many women expressed that menopause brings a type of freedom since they do not need to worry about pregnancy or menstrual mess when it comes to sexual activities^26,28,34,37,39^:

*‘I enjoy my sexuality at this stage of life. When I was young, I was scared to get pregnant’*. ^26^

For some women, getting older was synonymous with better sexual experiences as they had more information, more experience, more self-confidence, a better understanding of their sexual needs, a new partner, and/or felt less modest about sex^26,28,30-32,34,37,39,40^. Another sexual freedom expressed was that of menopause as a new stage of life where sex was no longer a priority and the relationship with their partner developed into something else, which they did not perceive as less satisfactory^30,39-43^:

*‘I am already old, and so is my husband, we are not concerned’*. ^42^

Sexual freedom was also voiced by women who could now say no to their husband’s sexual demands, though this was only a freedom in those cases where the husband accepted menopause as a valid excuse^23^.

### Rather no sex than bad sex

Low sexual desire, vaginal dryness, dyspareunia and difficulties in reaching orgasm were the most common sex-related symptoms of menopause expressed in these studies^23-39,41-43^. The women themselves reflected on how this easily became a vicious circle. When having sex was painful or took more effort, their desire to have sex decreased^23,30,33,39,43^. If a woman had a positive view of sex before menopause, she would look for a solution^25,28,30,32,43^, but if she had negative experiences to begin with, then giving up on sex was a more natural choice^23,24,31,34,36,41,43^. The struggle to reach orgasm also had a negative effect on women’s willingness to engage in sexual activities. Knowing that it would take a long time and require hard work made women think that it would not be ‘worthwhile’^30,31,38,39^:

*‘While I'm participating, I'm also feeling like, “Oh, why bother? It's not going to be as good. It's going to be much harder to do. This isn't going to happen for me”.’* ^39^

### It takes two

Women expressed that having a loving, communicative, supportive and understanding partner with whom they wanted to have sex, was a basis for finding a solution to or maintaining a continued sex-life during the menopausal transition^26,30,32,33,35,39,40,43^. An important aspect was sexual communication; in relationships where there was a joint desire to keep having sex, women expressed that adjusting and changing how they had sex, such as taking more time, engaging in more non-penetrative sex, using lubrication, sexual aids, or different positions made it possible to find ways together to keep enjoying sexual intimacy^30,38,39,41,43^:

*‘I will talk to him because of vaginal dryness and discomfort. I will tell him what to do, he will discuss with me what position we should take and when we [make love], it feels better’*. ^43^

Some women indicated that sex became a topic of discussion because of dyspareunia due to menopause^23,43^. Women who felt that their partners listened, communicated that they felt empowered, cared for, and encouraged to find a solution, or increased their sexual motivation^25,32,33^. For the women who expressed a lack of support, communication, or understanding from their partners, sex became a source of sorrow or conflict^23-25,31,33,34,41,43^:

*‘I have to obey him …My husband asks for [making love] every day … we have been using some lubrication and the [vagina] does not feel particularly dry … Just like cooking and taking care of the kids, it is something I have to do!’* ^43^

Many women communicated that the discrepancy between their own sex drive and their partner’s had a big impact on their sexual experience. Although the men usually had a higher sex drive^23-25,30-33,35,36,41,43^, there were also women who wanted more sex^24,25,28,31,43^. Some women feared the effects that menopause might have on their sexual relationships so much that they tried to prevent them, even if they did not yet have any problems^25^, while others hid their menopausal transition from their partners^23,26,29,34^.

### Privilege matters

Several sources of privilege were identified: having or not having access to time, space, money, knowledge, support and treatment, and autonomy. Some women expressed that it was hard to know if menopause caused their negative sexual experience or if it was due to life circumstances^32,36^:

*‘I don’t feel in the mood for sex, but I think it’s due to my surroundings more than my physical condition … My mother-in-law is living at home’*. ^30^

Privilege connected to money was seen in studies where efforts to maintain a youthful and slim appearance were expressed; the women adjusted according to their budgets, from creams and diets to plastic surgery^23,27,33-35,42^. Money was also an issue connected to medical visits and treatments, and lack of funds prevented women from seeking healthcare^27,29,41^.

Women’s experiences of their sexual autonomy differed in several ways. Some women communicated that since they grew up without sexual education or with strong stigmas surrounding female sexuality, their view on sex had been very restricted^23-26,30,32-34,37,38,41^. In certain contexts, women expressed that sex was a marital duty, due to a patriarchal society or religion, and therefore their sexual autonomy was not relevant^23-26,33,36,41,43^:

*‘I’m socially obliged to stay with my husband … in my family divorce is ominous. Despite the loss of my sexual motivation, I tolerate harsh sexual life conditions to preserve my marital life after 30 years of marriage.’* ^33^

### Support requires knowledge, will, access and acknowledgment

Women who mentioned healthcare support, did so primarily in the sense of general menopausal support rather than directly connected to sexual health. Diverse views were expressed about support from their healthcare systems, and several barriers where identified. To begin with, their view of menopause was essential; some women saw it as natural and something that could not be treated with medication^35,36,42^, while others saw it as a disease ^23,26,30,34,36^:

*‘When they saw my laboratory test, they said “Oh, my God, you have the menopause?”. It’s as if they were talking about cancer.’* ^23^

A few women suggested that menopausal healthcare should be subsidized since lack of funds prevented women from getting help^41^. Lack of trust in the healthcare system^23,26,29,37^ and lack of information in their native language^37^, were expressed as obstacles to accessing healthcare. These barriers were especially prevalent in the experiences of immigrant women compared to women in their country of residence, but also in comparison to women in their country of origin, women from low-income households compared to women from high-income households, women from rural areas compared to urban areas, and between self-identified African American, Chinese American and Mexican American women compared to self-identified European American women in the USA^29,37,42^:

*‘If you call the nurse and leave a message, she’ll call you back and sit on the phone with you and they’ll talk to you and walk you through stuff, you know?’* (European American, middle class)^29^

Stigmas around menopause and female sexuality were communicated in several articles, inhibiting women from seeking help and from turning to friends and family for support^23,25,36,38,41,42^. There were different underlying reasons for seeking treatment. Some women stated that they wanted support and treatment for menopausal symptoms because they wanted to have sex more tolerable^38,42,43^. For women who enjoyed sex and wanted to continue to do so, seeking support and help was seen as a natural solution^25,28,32,35,43^, though a few expressed frustrations over the limited treatment options available^38^:

*‘Why can’t they come up with a female Viagra? Something that improves sexual response in the same way you can help a man get an erection. Can't a woman, you know, feel more aroused. I remember the days I could think about sex, and I would start feeling tingly down there, you know?’* ^38^

Several women worried about possible side effects of MHT, such as cancer. The lack of information and support led to misconceptions and fear, preventing women from seeking help^28,30,35-38^. Many women communicated a need for support and empathy from partners, peers, colleagues, family or healthcare professionals^23,24,26,35-38,41,42^. Many also expressed that they wanted more knowledge, and some explicitly said that the information about sexuality in menopause was scarce^25,29,35-37,41,42^. A few women suggested that information be spread to the general public so that their partners would also receive the information^37,41^:

*‘I think it [information] should be more focused on sexual life. Because there is a lot of other information already … like there are videos about hot flashes, etc. … But not much about sex for both partners’*. ^41^

## DISCUSSION

The findings of this meta-synthesis highlight the complex and multifaceted experiences of women’s sexuality during the menopausal transition. Consistent with previous research, the participants’ narratives revealed a wide spectrum of experiences, shaped by biological changes, relational dynamics, social norms, and access to healthcare^9,47^. The overarching theme that ‘sexuality during menopause is not a homogenous experience’ underscores the importance of individualized approaches in both clinical practice and research.

The lack of adequate support from healthcare providers aligns with previous research stating a range of needs from society related to women’s sexual health: access to comprehensive sexual education including menopausal knowledge^48^, access to sexual and reproductive health and rights (SRHR)^49^, access to comprehensive menopausal healthcare^11^, and a society that acknowledges women as individuals with sexual desires, rights and autonomy^49^. The findings also resonate with the broader discourses surrounding menopause, which tend to frame it either as a medical condition requiring treatment or as a natural life stage that should be endured without intervention. The results indicate that both these perspectives risk overlooking women’s subjective experiences and healthcare needs. Women in societies with a strong medicalized view of menopause were more likely to think of it as an illness, or as the beginning of death. Women in societies viewing menopause as natural were encouraged to ‘endure’ this normal part of life. Hickey et al.^50^ state that the medicalization of menopause is problematic due to potential risks with MHT. Instead, they emphasize that menopausal care should focus on empowering women in this natural transition with restricted medical intervention. But a growing body of evidence suggests that the life-long positive health effects of MHT should be grounds for increased prescription, highlighting that focusing on the small risks reduce women’s quality of life^12,14,21,22^.

This meta-synthesis revealed that while some women embrace menopause as a liberating stage of life, others experience significant distress due to symptoms such as vaginal dryness, pain during intercourse, and loss of sexual desire. The availability of MHT could alleviate these symptoms and improve women’s quality of life. Leading menopausal guidelines shows that, when initiated within 10 years of menopause, MHT offers significant benefits for symptom relief and long-term health, outweighing any risks for the majority of women^18-20^.

However, despite clear guideline recommendations, the use of MHT remains low^51,52^. The recent Swedish nationwide survey by Götze Eriksson et al.^12^ provides valuable insights into the underlying reasons. The study found significant knowledge gaps among general practitioners and gynecologist, including misconceptions about indications, contraindications, and treatment duration. Importantly, the survey showed that over 90% of general practitioners mistakenly viewed a family history of breast cancer as a contraindication for MHT, a misconception not supported by the Swedish guidelines^20^. This suggests a persistent fear of cancer that is rooted in outdated interpretations of earlier studies, such as the Women’s Health Initiative trial^53^, despite subsequent re-evaluations that clarified these risks^54^.

The confusion surrounding local estrogen therapy further exemplifies knowledge gaps. Many women in this study reported pain during intercourse – symptoms typically associated with GSM – yet few mentioned using local estrogen treatment. Local estrogen, which acts directly on the vaginal tissues, is effective for treating GSM and is considered safe for long-term use^4,17^. According to Chism et al.^55^, one reason for the limited usage may be that the fear of breast cancer is so tightly connected to the word estrogen and in many countries the information labels for vaginal estrogen incorrectly state breast cancer as a risk.

For those women expressing sexuality as a high priority and requesting solutions regarding desire, dryness and ability to orgasm, testosterone has been shown to positively affect female libido. However, it is only prescribed off-label in some countries and licensed in even fewer^56^.

Our findings reveal that women seldom sought or received healthcare support explicitly targeting sexual health, despite navigating significant changes in sexual function during menopause. Instead, support was described more generally in relation to menopause. This absence of direct sexual healthcare support may stem from dual silence: on the one hand, women may feel discomfort or reluctance in raising these concerns; on the other hand, healthcare providers may fail in initiating such conversations. This goes in line with the ‘two-way taboo’ described by Graugaard^9^, where both parties assume the other is unwilling or unprepared to engage. This mutual hesitance is reinforced by stigma surrounding female sexuality in midlife and beyond, contributing to normalization and underreporting of sexual difficulties^47^.

Beyond individual-level barriers, systemic shortcomings such as insufficient sexual health training for healthcare professionals (HCPs) and the lack of integrated menopausal healthcare in their education, could further contribute to the invisibility of sexuality in clinical encounters^57,58^. Women may therefore encounter dismissive attitudes or generalized treatment recommendations that fail to address the nuanced impacts of menopause on intimacy. These patterns reflect missed opportunities for early intervention and holistic care. Addressing this gap requires both systematic and interpersonal change: integrating sexual health as a standard component of menopausal consultations, and equipping providers with communication tools to create safe, non-judgmental spaces where women feel validated in expressing sexual concerns. Barber and Charles^57^ identified barriers at each point in the menopausal healthcare journey. These included women not feeling empowered to seek medical advice, having their symptoms dismissed by HCPs, and encountering HCPs lacking knowledge or confidence to recognize the complexity of menopause and offer appropriate treatment or specialist referrals if outside their scope.

Furthermore, participants in our study demonstrated a lack of autonomy and the foundational conditions needed for making informed choices about their healthcare. Informed choice has many definitions^59-61^, but the core lies in adequate initial education and knowledge, the elimination of misinformation and myths, and easy access to healthcare and support. Informed choice is not about giving consent but rather having access to unbiased information and support so that any choice made belongs to the woman. Expanding menopausal healthcare beyond doctors and gynecologist to other allied healthcare professionals could be a way forward in meeting women’s needs of information and healthcare support.

Gender inequalities were highlighted in patriarchal and religious societies with intersecting systems of power dynamics where gender norms and standards, religious and cultural beliefs, marital (and familial) dynamics, and economic dependence influenced women’s sexual experiences during their menopausal transition. Women in these societies who expressed a lack of sexual autonomy described sex as a chore or a duty. Judith Butler's theory of gender performativity^62^ can be utilized to explore these women’s experiences of their sexuality, where it is part of their role as wives, and is something to get done rather than be enjoyed. These women’s gendered performance included being penetrated, and they experienced intercourse as ‘the real sex’. It is rare for women to be able to reach orgasm from penetrative sex^63^, and with GSM, many women described an increased discomfort or dyspareunia from it.

Women also expressed problems connected to discrepancies in desire between them and their partners. According to van Anders et al.^64^, women’s desire is often considered low because it is compared to their (male) partner’s sexual desire, a source of conflict that many women expressed in our study. van Anders et al.^64^ suggest that a heteronormative view, where women are sexually passive and men sexually active, has shaped empirical research on the topic. They challenge this perspective in the heteronormativity theory of low female sexual desire by encouraging researchers to consider gender norms and the gendered power order as an important contributing factor to perceived low desire in women partnered with men. As highlighted by Nagoski^8^, female sexual desire is often responsive rather than spontaneous, and the traditional framing of libido as either ‘high’ or ‘low’ can obscure women’s true needs and preferences. According to Philpott et al.^65^, sexual pleasure is a blind spot in research on sexuality, in the SDGs and in The Guttmacher-Lancet Commission on SRHR.

Our findings align with those of Wood et al.^6^ in acknowledging the broad societal and structural barriers faced by women during menopause, including inadequate access to information and persistent stigma. However, by focusing specifically on sexuality and healthcare interactions, our synthesis deepens the understanding of how these broader challenges manifest in intimate and relational domains. The present review expands on the Wood et al.^6^ framework by highlighting specific needs around sexual health education, bodily autonomy, and the role of gendered norms in clinical encounters.

### Strengths and limitations

The synthesis is based on 21 articles, reflecting the experiences of 610 women from 15 countries across various income levels. The recurring themes are likely relevant to women in similar contexts. The search and analysis process followed PRISMA guidelines^66^, enhancing methodological transparency. Recurrent themes across studies strengthen the reliability of the findings^67^. Despite varied perspectives on sexuality, the authors noted saturation, indicating consistent experiences^66^. Joint reading and coding of articles reduced bias and improved the accuracy of conclusions^67^. Efforts were made to minimize bias and approach the material with openness.

The study has some limitations. One limitation of the study is that only one article included non-heterosexual participants, leaving gaps in understanding sexuality when gender norms and penetration are less central. Another limitation is that menopausal status was not clearly defined in all articles (Table 2). However, these articles were included due to age of participants and symptom relevance, acknowledging that many women may lack the knowledge to self-assess their menopausal status. Meta-synthesis poses challenges, including language and time constraints, and the potential for missed articles not indexed in search engines^44^. Although searches combining menopause and sexuality yielded a manageable number of hits, additional insights might exist in broader studies on menopause.

## CONCLUSIONS

The complexity of menopause – encompassing biological, psychological, relational, and sociocultural dimensions, demands that women receive unbiased information and access to diverse treatment options tailored to their individual preferences and needs. By highlighting the concept of informed choice, this research will provide valuable insights into how healthcare systems can better facilitate patient-centered care, enabling women to navigate their menopausal transition with confidence and autonomy. This synthesis underscores the critical need for enhanced education and interdisciplinary collaboration within healthcare systems. Additionally, involving midwives, counsellors, and other allied healthcare professionals in menopause care could further support women’s diverse needs. Building on these findings, future research should focus on how to effectively support informed choice in menopausal healthcare.

## Data Availability

Data sharing is not applicable to this article as no new data were created.
